# Physical activity, return to work self-efficacy, and work status among employees undergoing chemotherapy for cancer - a prospective study with 12 months follow-up

**DOI:** 10.1186/s12885-021-07824-6

**Published:** 2021-02-17

**Authors:** Rikke Rosbjerg, Robert Zachariae, Dorte Gilså Hansen, Inger Hoejris, Saskia Duijts, Nina Lykkegaard Gehr, Irene Dyhrberg Andersen, Merete Labriola

**Affiliations:** 1 Department of Public Health, Aarhus University, Aarhus, Denmark; 2 DEFACTUM, Central Denmark Region, Denmark, P.P. Ørums Gade 11, 1.B, 8000 Aarhus, Denmark; 3 Unit for Psychooncology and Health Psychology, Department of Psychology, Aarhus University, Aarhus, Denmark; 4 Research Unit of General Practice, Institute of Public Health, University of Southern Denmark, Odense, Denmark; 5 Department of Oncology, Aarhus University Hospital, Aarhus, Denmark; 6 Amsterdam UMC, Vrije Universiteit Amsterdam, Department of Public and Occupational Health, Amsterdam Public Health research institute, Amsterdam, The Netherlands; 7 Department of Health and Care, Viby-Hoejbjerg, Aarhus Municipality, Aarhus, Denmark; 8 NORCE Norwegian Research Centre AS, Bergen, Norway; 9 Centre for Social Medicine, Frederiksberg and Bispebjerg Hospital, Copenhagen, Denmark

**Keywords:** Physical activity, Self-efficacy, Work status, Return to work, Cancer

## Abstract

**Background:**

Numerous studies emphasize the positive effects of physical activity on health and well-being in cancer patients. The effects of physical activity on the working lives of cancer patients have received less attention. The aim of the present study was to examine the association between physical activity and work status in employees with cancer, and the mediating role of return to work self-efficacy (RTWSE) in this association.

**Methods:**

Data from questionnaires (physical activity, RTWSE, performance status, sociodemographic), patient records, and Danish national registries (work status, education) were collected for 217 employees initiating chemotherapy for cancer. The associations of physical activity at baseline with work status at baseline and at twelve months follow-up, respectively, were estimated with logistic regression. The mediating role of RTWSE was investigated using the Sobel Goodmann test.

**Results:**

Employees with moderate (> 30 min/day) or high (> 150 min/day) levels of current daily activity at baseline had significantly increased odds for working at baseline (OR = 2.83, 95%CI = 0.73–10.96 and OR = 6.13, 95%CI = 1.68–22.40, respectively) and at twelve months (OR = 3.90, 95%CI = 1.19–12.77 and OR = 3.43, 95%CI = 1.12–10.51, respectively), compared to sedentary employees. Likewise, employees, physically active in their leisure time (light or vigorous psychical activity) for 2–4 h/week or > 4 h/week of light activity at baseline, had increased odds for working at twelve months (OR range = 1.20 (95%CI = 0.40–3.61)–5.39(95%CI = 0.78–37.32)), compared to sedentary employees. RTWSE was not found to mediate the observed associations.

**Conclusions:**

Physical activity appears positively associated with work status in employees undergoing treatment for cancer in the twelve months period after initiating chemotherapy.

## Background

Physical activity (PA) has been shown to be beneficial for the health and well-being of cancer patients [1, 2]. In addition to increasing physical function [2–4], PA during and after cancer treatment has been found associated with improved psychological functioning [2], increased quality of life [3, 5], reduced fatigue [4, 6, 7] and an increased sense of control [8]. The possible association between PA and work status of cancer patients has, however, received less attention.

Every year, 4.2 million individuals in Europe are diagnosed with cancer [9] of whom approximately 50% is at the working age [10, 11]. A substantial proportion of the occupationally active cancer patients experience difficulties in sustaining work or in returning to work during or after cancer treatment. They have more sick days, reduced work ability, lower productivity, and greater risk of early retirement compared with the general population [10, 12–15]. Furthermore, in a meta-analysis of 36 controlled studies, it has been found that patients with current or previous cancers are 1.4 times more likely to be unemployed than cancer-free controls [11]. Due to increasing cancer incidence [9] and substantial progress in cancer treatment, the number of cancer patients of working age is steadily increasing. This has led to a stronger demand for occupational rehabilitation for this group [16].

Including PA in rehabilitation programs to improve return to work (RTW) and work ability of employees with cancer has received increased interest within epidemiological research during the recent decade [17–19]. Some controlled efficacy trials have shown that patients with cancer participating in PA intervention programs RTW earlier [4, 20], for more hours [20, 21], and experience fewer problems at work, once back [22], than controls. In line with these findings, positive associations have been found in longitudinal observational studies between PA and RTW [23, 24]. However, in other controlled studies [25–28], no effects were found of multidisciplinary programs including PA on number of sick days and employment status, measured at follow-ups at three [26], six [28], or twelve months [25, 27]. Thus, the evidence regarding the associations between PA and work status in patients with cancer remains inconsistent and limited. Moreover, little is known about the mechanisms involved in the possible association between PA and work, i.e., why PA may be beneficial for RTW and work ability in patients with cancer.

A psychological factor shown to be of considerable importance in the RTW process is self-efficacy (SE) [29–31]. SE refers to the *“beliefs in one’s capabilities to organize and execute the courses of action required to produce certain attainments”* [32]. SE is situation specific and return to work-SE (RTWSE) has repeatedly proved to be predictive of actual RTW, and further, to be positively associated with work status and work ability in employees on sick leave due to both somatic and mental disorders [29, 31, 33–37]. Similar results have been observed in cancer populations [38, 39]. SE (more specifically general state SE and exercise SE) has furthermore proven to be positively associated with the level of PA, both in non-cancer [32, 40–42] and cancer populations [43, 44]. Being positively associated with both work (e.g., RTW, work ability, and work status) and PA, SE may play a mediating role [45] in the possible association between PA and work status. The hypothesis is that being physically active may increase RTWSE which may further affect the RTW or the work status of the cancer patient positively. This hypothesis has found support in two qualitative studies, in which “*increase in self-confidence*” [46] and “*increase in the confidence in physical abilities*” [19] were reported as positive influences of PA on work. However, to the best of our knowledge, the mediating role of RTWSE in the possible association between PA and work status has not yet been examined.

The primary aim of the present study was to examine the association between PA and work status in employees undergoing chemotherapy for cancer, and furthermore, to examine the mediating role of RTWSE in this association. This was investigated in a design combining cross-sectional and longitudinal analyses examining: I) the association between PA and work status at baseline; II) the association between PA, reported at baseline, and work status at twelve months after baseline; and III) the mediating role of RTWSE, measured at three months, in the possible association between PA, reported at baseline, and work status at twelve months after baseline.

## Methods

### Study design and setting

The study population included employees with various cancers initiating chemotherapy at Aarhus University Hospital, Denmark, between November 2016 and May 2018, who were invited to participate in a longitudinal survey regarding PA and work life [47]. The participants of the survey were asked to complete questionnaires at initiation of chemotherapy (baseline) and at three, six, and twelve months after baseline. A previous study examining the predictive value of RTWSE on actual RTW was based on the same study population [47].

In the present study, the data sources included patient questionnaires completed at baseline and at three months, data from patient records obtained at baseline, and data from Danish national registries obtained at baseline and at twelve months. The STROBE guideline for cohort studies guided the design, the analyses, and the presentation of the present study [48].

### The Danish sick leave policy

The RTW rates of employees with cancer are greatly influenced by the sick leave policy of the given country [13, 49]. In Denmark, all members of the work force are entitled to receive sickness absence compensation from the municipality after four weeks of sickness absence [50]. Receiving sickness absence compensation is possible for 22 weeks for all citizens. The sickness absence compensation period can be prolonged for citizens with a severe, life-threatening illness, i.e., extension is possible for many cancer patients. If the employee receives salary as usual during the sickness absence period, the employer is entitled to receive the compensation.

### Participants

#### Inclusion criteria

In the survey, patients were considered eligible based on the following inclusion criteria: I) age 18–62, II.a) initiating chemotherapy for a newly diagnosed cancer disease, or II.b) due to relapse, if the patient had not initiated chemotherapy during the last 24 months, III) all treatment intentions (i.e., curative, palliative, adjuvant, and neo-adjuvant), IV) having an employment contract at the time of inclusion (working, on full, or on part time sick leave), and V) ability to read and understand Danish [47].

#### Procedure

At the first chemotherapy session, eligible patients with regard to age and history of cancer were introduced to the study by a clinical nurse. If the nurses, during the first chemotherapy session, considered an eligible patient to be incapable of receiving additional information beyond treatment-related information, they postponed the introduction to the study until the second or third chemotherapy session. Patients interested in learning more about the study signed a contact sheet which allowed a research assistant to contact the patients by telephone. On the phone, the patients were screened regarding employment status by a research assistant and those who were eligible and wanted to participate signed a written informed consent. Subsequently, the baseline questionnaire was sent to the patients by e-mail or regular mail in accordance with the patient’s preference. At three months, a similar questionnaire without the demographic items was distributed. Two reminder e-mails were sent after five and ten days, respectively, in case of no response [47]. The procedure is described in detail in Rosbjerg et al. [47].

#### Study population

**Fig. 1:**
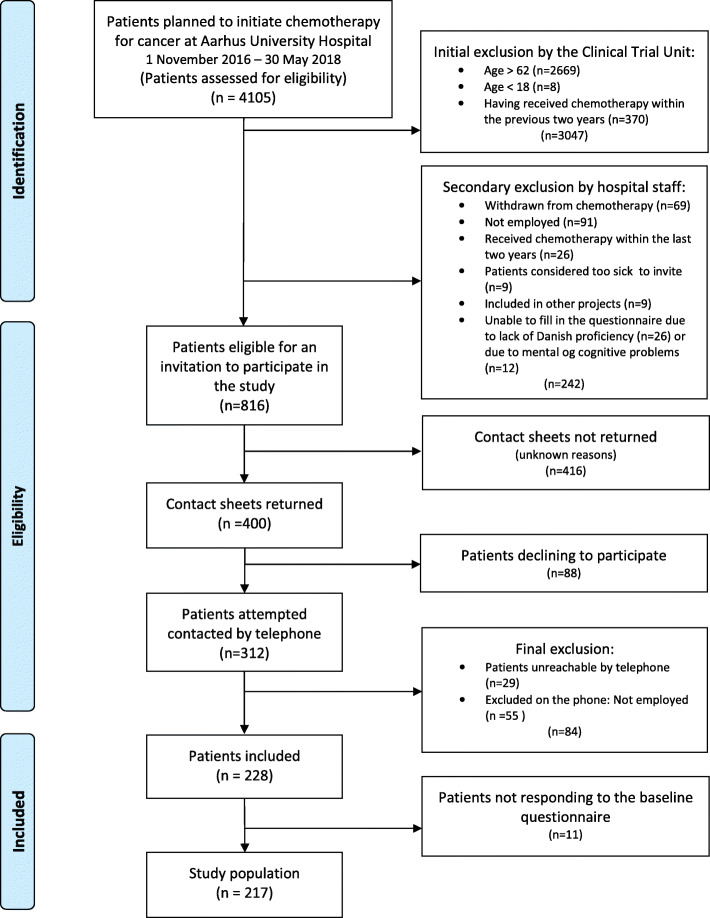
Flow chart of inclusion

During the inclusion period from November 2016 – May 2018, a study population of 228 patients was reached. However, eleven did not return the baseline questionnaire, leaving a baseline population of 217 patients (Fig. 1) [47].

### Variables of interest

#### Dependent variable

##### Work status

Information regarding work status at baseline and at twelve months was obtained through the “Danish Register for Evaluation of Marginalization” (DREAM). DREAM is a Danish registry which contains data on all public transfer payments which have been administered to Danish citizens since August 1991, e.g., sickness absence compensation, early retirement, etc.. The transfer payments are registered for each citizen on a weekly basis if the person receives public transfer payments for one day or more per week. If there is no transfer payment registered, the citizen is rated as self-supported. DREAM is considered to be a valid tool for follow-up studies of RTW and sickness absence [51, 52]. The primary dependent variable was work status at baseline and at twelve months after baseline, defined in DREAM categories as: I) “at work” (including both full time working and part time sick leave), and II) “not at work” (including any kind of sickness absence compensation, permanent exit from the labor marked (i.e., retirement), and death (i.e., those who died in the 12 months follow-up period). The two last mentioned categories were only possible at the twelve-month follow-up, as all participants were alive and employed at time of inclusion).

Each participant was thus followed in the DREAM database for twelve months after his/her inclusion. However, the participants who died during follow-up were not followed for twelve months. They were included among those who were not working at twelve months, i.e., the dependent variable “not at work”.

#### Independent variables

The independent variables were measured at baseline and at three months follow-up, except for the sociodemographic variables which were measured only at baseline.

### Physical activity (PA)

PA was measured by three variables: *The pre-illness level of leisure time PA*, *the current level of leisure time PA* and *the current level of daily PA*. The first-mentioned was measured at baseline only.

### The pre-illness level of leisure time PA (Pre-illness PA_leisure_)

Pre-illness PA_leisure_ was defined as the level of PA during the twelve months prior to the cancer diagnosis (the current cancer diagnosis in case of relapse patients) and was measured by the Danish version of *The Saltin-Grimby Physical Activity Level Scale* [53]*,* a four level scale of leisure time PA. The scale has been shown to have good concurrent validity [54], including in Denmark [55]. Based on the following recommendation by Grimby [54]: “*Mixing two intensities of activity within one activity level is not to recommend and makes the interpretation of the results difficult”*, the original level III “low intensity PA >4 hours or vigorous PA for 2–4 hours/week” was spilt into two levels, i.e., level III and IV. The scale thus consisted of five levels: I) sedentary or low intensity PA < 2 h/week; II) low intensity PA 2–4 h/week; III) low intensity PA >4 h/week; IV) vigorous PA for 2–4 h/week; and V) vigorous PA >4 h/week.

### The current level of leisure time PA (current PA_leisure_)

Current PA_leisure_ was measured by use of the Danish version of *The Saltin-Grimby Physical Activity Level Scale* [53, 54] as well, in which the participants were asked to categorize themselves according to one of the five above mentioned categories based on their own level of PA for the last seven days.

### The current level of daily PA (current PA_daily_)

Current PA_daily_ was measured by the Danish version of *The International Physical Activity Questionnaire, long version (IPAQ-long)* [56], a measure of self-reported PA validated in several countries [57], including Denmark [58]. The questionnaire consists of 15 items and measures PA in four domains: work, transportation, housework/gardening and leisure time. Thinking of the past seven days, the respondent is asked to report duration (i.e., hours and minutes) and number of days of PA during the past seven days in all four domains and within each domain at three different levels (i.e., low, moderate, and high). Days and time spent on PA in each level in each domain are converted into MET (Metabolic Equivalent of Task)-minutes/week and are hereafter, according to the IPAQ guidelines, converted to total PA at either low (below 600 MET-minutes/week), moderate (600–3000 MET-minutes/week), or high (at least 3000 MET-minutes/week) level. 600 MET-minutes/week correspond to 30 min PA per day on average, which again corresponds to the recommended weekly level of PA across health boards. Covering four domains and hence the PA during all daily activities and not only leisure time, the IPAQ-measured level of PA was thus defined as the current *daily* level of PA.

### Return to work self-efficacy (RTWSE)

RTWSE was measured by the 19-item *RTWSE-19 questionnaire*, concerning a person’s belief in his or her own ability to handle different aspects of returning to work [29, 59]. Every item is scored on an 11-point numerical rating scale (“not at all certain” (0) to” completely certain”(10)). Adding all scores and dividing by the number of completed items calculates the mean score for the total scale. The total score thus ranges from 0 to 10. In the present study, the scale was dichotomized into low (≤7.5) and high (> 7.5) RTWSE according to the highest tertile as originally reported by Shaw et al. [29]. In the Danish validation of the questionnaire, Cronbach’s α value was 0.97 for the total score [59]. Total scores were categorized as missing in case of > 20% missing values, according to the guidelines [60].

### Performance status

The widely used *performance status scale* developed by The Eastern Cooperative Oncology Group was used to measure performance status [61]. The participants were asked to categorize themselves according to five levels of performance: 0) fully active, able to carry on all pre-disease performance without restriction; I) restricted in strenuous activity but able to carry out work of a light nature; II) capable of all self-care but unable to carry out any work activity, up and about for > 50% of the time; III) capable of only limited self-care, in bed for > 50% of the time, or IV) cannot carry out any self-care, totally confined to bed or chair.

### Sociodemographic and illness- and treatment-related variables

At baseline, the participants were asked to fill out information regarding age, gender, ethnicity, level of education, marital status and children living at home. Information regarding education was further obtained from the Danish Education Register of Statistics Denmark and categorized into four levels: I) none: < 10 years of education, II) short: 10–12 years of education, III) moderate: 13–15 years of education, and IV) long: > 15 years of education [62]. As in Rosbjerg et al. [47], if information regarding education were missing in the registry, the self-reported information regarding educational level was used. The participants further reported job type (sedentary, physical, mixed) and information on having leadership tasks (yes/no), being self-employed (yes/no) and perceived support from the workplace (on a 10-item rating scale with 10 indicating high level of perceived support). The following illness- and treatment-related variables were obtained from patient records at baseline: Type of cancer, time since diagnosis (days), time since initiation of chemotherapy (days), number of treatment modalities in addition to chemotherapy, and treatment intention (curative, palliative).

### Analysis

#### Descriptive statistics

Baseline data regarding sociodemographic and illness- and treatment-related variables as well as baseline levels of PA, RTWSE, and performance status were presented as frequencies and percentages, by means and standard deviations (SD) or by medians and interquartile ranges (IQR) and compared between the group of full time sickness absent participants and the group of working participants, using unpaired samples t-tests, Mann-Whitney U tests, chi-squared tests or Fisher’s exact tests.

#### Associations between PA and work status at baseline (objective I)

Using logistic regression, the Odds Ratios (ORs) were estimated for the associations of pre-illness and current level of PA, respectively, and work status at baseline. In model 1, unadjusted analyses were conducted. In model 2, the following sociodemographic variables were adjusted for: gender, age, and educational level. In model 3, the following illness- and treatment-related variable was added: treatment intention. In model 4, performance status was further added. The following categorical covariates were dichotomized in the multiple models: educational level (none/short versus moderate/long), and performance status (level 0 versus level ≥ 1).

#### Associations between PA at baseline and work status at twelve months (objective II)

Using logistic regression, the ORs were estimated for the associations of pre-illness and current level of PA, respectively, with work status at twelve months. Those who died during follow-up were categorized as “not working” at twelve months. In model 1, unadjusted analyses were conducted. In model 2, the following sociodemographic variables were adjusted for: gender, age, educational level, and baseline work status. In model 3, the following illness- and treatment-related variable was added: treatment intention. In model 4, performance status was further added. Baseline work status was added as a covariate in this model as previous research have shown that previous sick leave is negatively associated with work status [63]. The categorical covariates were dichotomized as described above.

#### The mediating role of RTWSE (objective III)

The Sobel Goodmann test was intended to be used to analyze the mediating role of RTWSE in the associations between baseline PA and work status at twelve months, using the three months level of RTWSE. These analyses were restricted to the cases of a statistically significant association between baseline PA and work status at twelve months (i.e., objective II). The Sobel Goodmann test was furthermore restricted to cases fulfilling the following preconditions to establish mediation: the independent variable (i.e., baseline PA) must significantly affect the mediator (i.e., RTWSE), and the mediator (i.e., RTWSE) must significantly affect the dependent variable (i.e., work status) [45]. These preconditions were tested using univariate logistic regression analyses.

#### Loss to follow-up

Due to non-response at three months (i.e., RTWSE, three months), a loss to follow-up of 14% (*n* = 30) occurred. No differences with regard to sociodemographic and illness- and treatment-related characteristics were found between responders and non-responders, except for ethnicity, i.e., significantly (*p* < 0.001) more non-Danish participants compared to Danish participants were loss to follow-up. At twelve months follow-up, solely data from the Danish national registry, DREAM, was included, resulting in 100% complete cases.

All analyses were performed using STATA 15.1 [64] and a 5% level of statistical significance.

## Results

### Descriptive statistics

At baseline, 135 participants (62%) were on full time sick leave while 82 participants (38%) were working. Baseline sociodemographic and illness- and treatment-related characteristics are shown in Table 1. The distribution of baseline characteristics did not differ significantly between sickness absent and working employees, except regarding leadership and being self-employed, i.e., significantly more leaders (*p* < 0.05) and significantly more self-employed (*p* < 0.01) were working at baseline compared to subordinates and salaried employees, respectively. At twelve months, 154 (71%) were working, 35 (16%) were on full time sick leave, eight (4%) were early retired and 20 (9%) had died. Of the 135 participants who were on full time sick leave at baseline, 85 (63%) had returned to work twelve month later.

**Table 1:** Baseline sociodemographic and illness- and treatment-related characteristics of a sample of employees undergoing chemotherapy for cancer, working/at part time sick leave or at full time sick leave at baseline. Mean and standard deviation, median and interquartile range, 95% confidence interval, frequency and percentage, and *p*-values

	Working / at part time sick leave (*N* = 82)	At full time sick leave (*N* = 135)	
	Mean (SD) / Median (IQR)	Mean (SD) / Median (IQR)	P-value
Age (years), mean (SD)	52 (7.10)	50 (7.34)	0.063
Missing	0	0	
Time since diagnosis (days), median (IQR)	71.50 (48–98)	72.00 (49–96)	0.900
Missing	0	0	
Time since initiation of chemotherapy (days), mean (SD)	32 (18.71)	34 (19.55)	0.536
Missing	0	0	
Perceived support from the work place ^a^, mean (SD)	9.23 (1.73)	8.57 (2.31)	0.051
Missing	20	11	
	N (%)	N (%)	
Gender			0.056
Female	52 (63)	102 (76)	
Man	30 (37)	33 (24)	
Missing	0 (0)	0 (0)	
Ethnicity			0.203
Danish	76 (93)	125 (93)	
Other	0 (0)	4 (3)	
Missing	6 (7)	6 (4)	
Educational level			0.425
None	7 (8)	12 (9)	
Short	40 (49)	53 (39)	
Medium	21 (26)	48 (36)	
Long	10 (12)	19 (14)	
Missing	4 (5)	3 (2)	
Work type			0.130
Physical	14 (17)	35 (26)	
Sedentary	42 (51)	53 (39)	
Mixed	20 (25)	41 (30)	
Missing	6 (7)	6 (5)	
Self-employed			**0.002**
Yes	13 (16)	5 (4)	
No	63 (77)	124 (92)	
Missing	6 (7)	6 (4)	
Leadership			**0.024**
Yes	21 (26)	19 (14)	
No	55 (67)	110 (82)	
Missing	6 (7)	6 (4)	
Marital status			0.851
Married	60 (73)	106 (79)	
Living with parents	0 (0)	0 (0)	
Widower	1 (1)	1 (1)	
Divorced	12 (15)	19 (14)	
Have always lived alone	3 (4)	3 (2)	
Missing	6 (7)	6 (4)	
Children living at home			0.569
No	41 (50)	59 (44)	
Yes	35 (43)	68 (50)	
Missing	6 (7)	8 (6)	
Type of cancer			0.645
Female reproductive system	3 (4)	8 (6)	
Breast	42 (51)	69 (51)	
Lung incl. Mesotheliomas	5 (6)	11 (8)	
Urological incl. Male reproductive system	8 (10)	5 (4)	
Upper gastrointestinal	8 (10)	13 (10)	
Colorectal	7 (8)	17 (12)	
Cerebral and the central nervous system	5 (6)	5 (4)	
Other	4 (5)	7 (5)	
Missing	0 (0)	0 (0)	
Treatment intention			0.333
Curative	59 (72)	105 (78)	
Palliative	23 (28)	30 (22)	
Missing	0 (0)	0 (0)	
Treatment modalities			0.864
Chemotherapy	64 (78)	109 (81)	
Chemotherapy and one additional treatment modality	16 (20)	22 (16)	
Chemotherapy and two additional treatments modalities	2 (2)	4 (3)	
Missing	0 (0)	0 (0)	

*SD* standard deviation

*IQR* interquartile range

^a^ = measured on a 10-point rating scale with 10 indicating high level of perceived support

No differences were found between working and sickness absent participants at baseline with regard to the pre-illness and current level of PA_leisure_, and performance status (Table 2). However, the working participants reported a significantly higher level of current PA_daily_ (*p* < .001), as well as a significantly higher level of RTWSE (p < .001) than the participants at full time sick leave at baseline (Table 2).

**Table 2:** Baseline measures of return to work self-efficacy, physical activity, and performance status in a sample of employees undergoing chemotherapy for cancer, working / at part time sick leave or at full time sick leave at baseline. Median and interquartile range, 95% confidence interval, frequency and percentage, and p-values

	N	Working / at part time sick leave	N	At full time sick leave	P-value
		Median (IQR), 95% CI, range		Median (IQR), 95% CI, range	
Return to work self-efficacy total scale	76	8.29 (6,92–9.39), 7.63–8.90,	131	6.95 (4.89–8.58), 5.89–7.72,	**p < .001**
		N (%)		N (%)	
Return to work self-efficacy	76		131		***P*** **< .01**
Low (≤7.5)		27 (36)		75 (57)	
High (> 7.5)		49 (64)		56 (43)	
Pre-illness leisure time physical activity	82		135		0.592
Sedentary		6 (7)		11 (8)	
Light activity 2–4 h/week		13 (16)		30 (22)	
Light activity >4 h/week		28 (34)		38 (28)	
Vigorous activity 2–4 h/week		30 (37)		43 (32)	
Vigorous activity > 4 h/week		5 (6)		13 (10)	
Current leisure time physical activity	82		135		0.068
Sedentary		11 (13)		36 (27)	
Light activity 2–4 h/week		35 (43)		50 (37)	
Light activity >4 h/week		22 (27)		31 (23)	
Vigorous activity 2–4 h/week		13 (16)		12 (9)	
Vigorous activity > 4 h/week		1 (1)		6 (4)	
Current daily physical activity	79		130		**p < .001**
Low		3 (4)		23 (18)	
Moderate		22 (28)		51 (39)	
High		54 (68)		56 (43)	
Performance status	75		130		0.499
Level 0: Fully active, able to carry on all pre-disease performance without restriction		22 (29)		31 (24)	
Level 1: Restricted in strenuous activity, but able to carry out work of a light nature		44 (59)		75 (58)	
Level 2: Capable of all self-care but unable to carry out any work activity		9 (12)		21 (16)	
Level 3: Capable of only limited self-care, in bed for > 50% of the time		0 (0)		3 (2)	
Level 4: Cannot carry out any self-care, totally confined to bed or chair		0 (0)		0 (0)	

*SD* standard deviation

*IQR* Interquartile range

*CI* confidence interval

### Associations between PA and work status at baseline (objective I)

As seen in Table 3, employees with a moderate (i.e., > 30 min/day on average) or a high level (i.e., > 150 min/day on average) of current PA_daily_, at baseline, had significantly increased odds for working at baseline (OR = 2.83, 95%CI = 0.73–10.96 and OR = 6.13, 95%CI = 1.68–22.40, respectively), compared to sedentary employees. This association remained significant (*p* = 0.010) when adjusting for age, gender, level of education, treatment intention, and performance status.

**Table 3:** Associations between baseline levels of physical activity and working at baseline in a population of employees undergoing chemotherapy for cancer. Odds Ratios, 95% confidence intervals, and p-values of the unadjusted and the multivariate logistic regression models

		Model 1 (unadjusted)				Model 2^a^				Model 3^b^			Model 4^c^			
Variable	N	OR	95% CI	*P* value	N	OR	95% CI	P value	N	OR	95% CI	P value	N	OR	95% CI	P value
Pre-illness level of leisure time physical activity	217			0.582	210			0.194	210			0.268	202			0.241
Sedentary		1.00	–			1.00	–			1.00	–			1.00	–	
Light activity 2–4 h/week		0.79	0.24–2.61			0.87	0.25–2.98			0.87	0.25–3.01			0.91	0.26–3.22	
Light activity >4 h/week		1.35	0.45–4.09			1.46	0.46–4.62			1.47	0.46–4.64			1.40	0.44–4.47	
Vigorous activity 2–4 h/week		1.28	0.43–3.84			1.42	0.46–4.39			1.44	0.46–4.51			1.32	0.42–4.18	
Vigorous activity >4 h/week		0.71	0.17–2.96			0.62	0.14–2.77			0.63	0.14–2.91			0.43	0.09–2.14	
Current level of leisure time physical activity	217			0.058	210			**0.014**	210			**0,024**	202			0.066
Sedentary		1.00	–			1.00	–			1.00	–			1.00	–	
Light activity 2–4 h/week		2.29	1.03–5.11			2.98	1.25–7.09			2.98	1.25–7.08			2.61	1.08–6.29	
Light activity >4 h/week		2.32	0.97–5.54			3.13	1.24–7.92			3.13	1.24–7.91			2.87	1.11–7.43	
Vigorous activity 2–4 h/week		3.55	1.26–9.98			4.45	1.45–13.63			4.44	1.45–13.59			3.42	1.02–11.49	
Vigorous activity >4 h/week		0.55	0.06–5.03			0.75	0.08–7.37			0.76	0.08–7.52			0.62	0.06–6.48	
Daily physical activity	209			**< 0.001**	202			**0.002**	202			**0.004**	194			**0.010**
Low		1.00	–	–		1.00	–	–		1.00	–	–		1.00		
Moderate		3.31	0.90–12.17			3.39	0.90–12.73			3.35	0.88–12.69			2.83	0.73–10.96	
High		7.39	2.10–26.06			6.99	1.95–25.11			6.93	1.92–25.00			6.13	1.68–22.40	

*OR* Odds Ratio

*CI* Confidence interval

^a^ Adjusted for gender, age, and educational level

^b^ Adjusted for gender, age, educational level, and treatment intention

^c^ Adjusted for gender, age, educational level, treatment intention, and performance status

When looking at the overall effect of PA in the leisure time, no significant associations were found in the unadjusted models between PA in the leisure time, pre-illness or current level, and work status at baseline. However, certain levels of current PA_leisure_ in the unadjusted model (model 1, Table 3) showed significant associations between current PA_leisure_ and work status, i.e., employees reporting 2–4 h of light (OR = 2.29, 95%CI = 1.03–5.11) or 2–4 h of vigorous (OR = 3.55, 95%CI = 1.26–9.98) activity weekly had significantly increased odds for working at baseline, compared to sedentary employees.

Likewise, certain levels of current PA_leisure_ in models 2 and 3 showed significant associations between levels of PA and work status, i.e., employees reporting 2–4 h light or vigorous PA_leisure_ or > 4 h light PA_leisure_ weekly had significantly increased odds for working at baseline compared to sedentary employees, when adjusting for gender, age, educational level, and treatment intention (Table 3).

### Associations between PA at baseline and work status at twelve months (objective II)

As seen in Table 4, employees with a moderate (i.e., > 30 min/day on average) or a high level (i.e., > 150 min/day on average) of daily PA at baseline, had significantly increased odds for working twelve months after baseline (OR = 3.90, 95%CI = 1.19–12.77 and OR = 3.43, 95%CI = 1.12–10.51, respectively), compared to sedentary employees. These associations remained statistically significant (*p* < 0.001) when adjusting for age, gender, level of education, baseline work status, treatment intention, and performance status.

**Table 4:** Associations between baseline levels of physical activity and working at twelve months in a population of employees undergoing chemotherapy for cancer. Odds Ratios, 95% confidence intervals, and p-values of the unadjusted and the multivariate logistic regression models

	Model 1 (unadjusted)				Model 2^a^				Model 3^b^				Model 4^c^			
Variable	N	OR	95% CI	P value	N	OR	95% CI	P value	N	OR	95% CI	P value	N	OR	95% CI	P value
Previous level of leisure time physical activity	217			0.253	210			**< 0.001**	210			**< 0,001**	202			**< 0.001**
Sedentary		1.00	–			1.00	–			1.00	–			1.00	–	
Light activity 2–4 h/week		2.06	0.60–7.10			2.57	0.68–9.68			3.67	0.84–16.02			3.87	0.84–17.74	
Light activity >4 h/week		1.35	0.44–4.17			1.65	0.49–5.60			1.66	0.44–6.30			1.67	0.44–6.41	
Vigorous activity 2–4 h/week		1.45	0.47–4.43			1.31	0.40–4.30			2.33	0.62–8.79			2.42	0.63–9.24	
Vigorous activity >4 h/week		0.55	0.14–2.12			0.82	0.18–3.75			1.71	0.31–9.47			1.74	0.31–9.85	
Current level of leisure time physical activity	217			**0.024**	210			**< 0.001**	210			**< 0.001**	202			**< 0.001**
Sedentary		1.00	–			1.00				1.00				1.00	–	
Light activity2–4 h/week		2.26	1.06–4.83			1.81	0.78–4.23			2.09	0.79–5.57			1.87	0.68–5.12	
Light activity>4 h/week		1.88	0.82–4.31			1.32	0.53–3.30			1.28	0.44–3.68			1.20	0.40–3.61	
Vigorous activity 2–4 h/week		5.43	1.43–20.70			5.06	0.99–25.78			5.58	0.92–33.79			5.39	0.78–37.32	
Vigorous activity >4 h/week		0.56	0.11–2.76			0.43	0.08–2.49			0.55	0.07–4.38			0.54	0.07–4.54	
Daily physical activity	209			**0.001**	202			**< 0.001**	202			**< 0.001**	194			**< 0.001**
Low		1.00	–			1.00	–			1.00	–			1.00	–	
Moderate		4.89	1.89–12.67			4.30	1.55–11.95			3.74	1.23–11.38			3.90	1.19–12.77	
High		4.92	2.00–12.12			3.98	1.48–10.67			3.52	1.21–10.22			3.43	1.12–10.51	

*OR* Odds ratio

*CI* Confidence interval

^a^ Adjusted for gender, age, educational level, and baseline work status

^b^ Adjusted for gender, age, educational level, baseline work status, and treatment intention

^c^ Adjusted for gender, age, educational level, baseline work status, treatment intention, and performance status

Likewise, employees who were physically active in their leisure time at baseline (i.e., current PA_leisure_), i.e., reporting 2–4 h of light or vigorous activity weekly, or > 4 h of light PA weekly, had increased odds for working twelve months after baseline (OR = 1.87, 95% CI = 0.68–5.12, OR = 5.39, 95% CI = 0.78–37.32, and OR = 1.20, 95% CI = 0.40–3.61, respectively), compared to employees who were sedentary in their leisure time when adjusting for gender, age, educational level, baseline work status, treatment intention, and performance status (Table 4). On the contrary, employees reporting vigorous activity in their leisure time for > 4 h/week at baseline had decreased odds for working twelve months after baseline (OR = 0.54, 95% CI = 0.07–4.54) compared to employees who were sedentary in their leisure time (Table 4). However, as seen in Table 4, only the overall effect of current PA_leisure_ was significantly associated with twelve-month work status. None of the individual levels of the variable were significantly different from sedentary behavior (current PA_leisure_, model 4, Table 4).

No significant associations between pre-illness PA_leisure_ and work status at twelve months were found, when looking at the unadjusted model (pre-illness PA_leisure_, model 1, Table 4). However, models 2, 3, and 4 showed an overall significant association between pre-illness PA_leisure_ and work status when adjusting for gender, age, educational level, baseline work status, treatment intention, and performance status (*p* < 0.001) (Table 4).

### The mediating role of RTWSE (objective III)

**Fig. 2:**
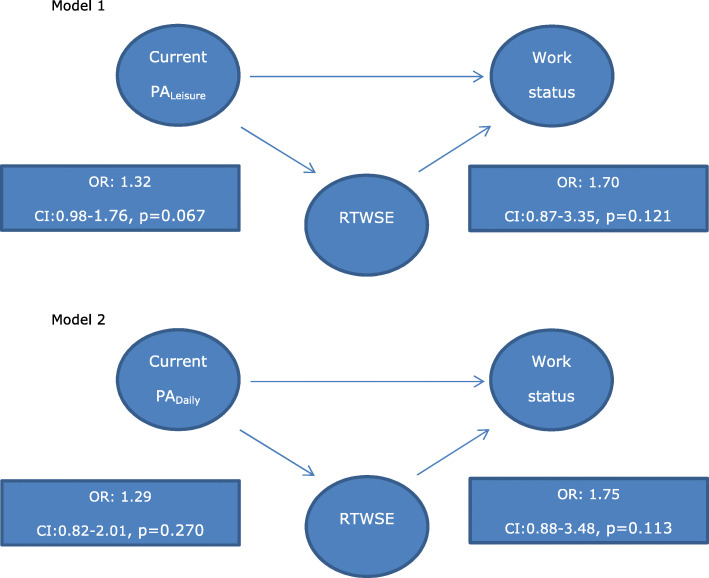
Associations between Current leisure time physical activity, measured at baseline, and Return to work self-efficacy, measured at three months and between Return to work self-efficacy, measured at three months, and work status at 12 months (model 1) and associations between Current daily physical activity, measured at baseline, and Return to work self-efficacy, measured at three months, and between Return to work self-efficacy, measured at three months, and work status at twelve months (model 2). PA: Physical activity. RTWSE: Return to work self-efficacy. OR: Odds Ratio. CI: Confidence Interval

The mediating role of RTWSE was examined based on the significant associations between current PA_daily_ and work status at twelve months and current PA_leisure_ and work status at twelve months, respectively (i.e., objective II). However, the preconditions of RTWSE being a mediator in these associations were not fulfilled, as RTWSE was not significantly associated with neither the independent variable (PA) nor the dependent variable (work status) in either of the models (Fig. 2). The hypothesis of RTWSE being a mediator between PA and work status was thus rejected and hence, the Sobel Goodmann test was not conducted.

## Discussion

### Main findings

Based on a sample of 217 employees receiving chemotherapy for various cancers, the present study supports the hypothesis of a positive association between PA and work status over a twelve months follow-up period. However, no support was found for a mediating role of RTWSE. To our knowledge, the present study is the first testing the hypothesis of RTWSE being a mediator in the association between PA and work status. Of the 135 participants being on full time sick leave at baseline, 85 (63%) had returned to work at twelve months. This RTW rate resembles previously reported international RTW rates of cancer patients, i.e., on average 62% (range 30–93%) one to two years after diagnosis [10, 14].

### Interpretation of findings and implications

#### Associations between PA and work status at baseline (objective I)

Of employees initiating chemotherapy for cancer, employees with a moderate to high level of daily activity, i.e., > 30 min/day in average, were more likely to be working than sedentary employees. The causal relationship is, however, not possible to conclude upon due to the cross-sectional design. It is possible that the working employees reported higher levels of PA_daily_ simply due to their physically active working hours and PA during transportation to work. Furthermore, the observed association could also be explained by other potentially confounding factors. Yet, adjusting for predefined independent variables in the logistic regression models was a way of minimizing the risk of potential confounders [65], i.e., age, gender, level of education, treatment intention, and performance status.

#### Associations between PA at baseline and work status at twelve months (objective II)

Following the participants for twelve months, it was found that the employees with a moderate or high level of daily activity at initiation of chemotherapy, i.e., > 30 min/day in average, were significantly more likely to be working at twelve months follow-up, compared to sedentary participants.

Similarly, an overall significant positive association between PA_leisure_ at baseline and work status was found, i.e., the employees who were physically active during their leisure time at initiation of chemotherapy were more likely to be working at twelve months as well. Interpreting these results, it should, however, be kept in mind, that the individual levels of activity were not significantly different from sedentary behavior, thus indicating a less secure association between the individual levels of activity and work status than between the variables PA_leisure_ and work status in general. Using an alternative questionnaire for measuring PA_leisure_ might yield different results regarding the significance of the individual levels of activity. Furthermore, contradictory to the hypothesis of a positive association between PA and work status, the participants reporting > 4 h of vigorous PA during leisure time showed decreased odds for being at work at twelve months after baseline. This may be explained by a motivation of these participants to spend their energy on their sports and leisure activities rather than at work. The role of motivation in the RTW process among cancer patients has been underlined in reviews regarding the RTW process of cancer survivors [12]. Yet, the number of participants at the level of “vigorous activity >4 hours/weekly” is small (i.e., *n* = 7 (3%), see Table 2) and therefore, the specific estimates of this level of activity should be interpreted with caution. Generally, the CIs in the multivariate models in this study are large, indicating lack of precision of the estimates. Large CI’s can be explained by a small sample size [65]. Larger study samples are recommended in the future to ensure more participants at all activity levels and thereby a greater precision of the estimates.

Despite the contradictory results of the activity level “vigorous activity >4 hours/weekly”, the results of the present study generally support the hypothesis of a positive association between PA and work status of cancer patients. Other observational studies have found support for the positive effect of PA on work status as well. In an observational design [24], it was found that women with breast cancer who participated in exercise/sports at time of diagnosis were more likely to RTW within three years. Likewise, in an observational design as well, positive effects of a PA intervention program on RTW during 18 months of follow-up in a population of cancer patients were reported [23]. However, even with follow-up data, inferring causation requires a randomized controlled trial (RCT) [66]. In a matched case-control design [21], it has been shown that patients with cancer participating in a high-intensity exercise program minimized the decrease in work ability after their cancer diagnosis, measured three years after diagnosis, compared to an age-matched control group of cancer patients from two other hospitals receiving care as usual. In RCTs, beneficial effects of PA intervention programs on RTW [4, 20] and work ability [20, 22] were found, but in these studies the work-related variables were not examined as the primary outcome measures. Other RCTs have rejected the hypothesis of a positive effect of PA on RTW and work ability [25–28]. However, these studies included a pilot study [26] and a feasibility study [28], both with few participants (*n* = 41 and *n* = 18, respectively), and two studies that included light PA programs, relaxation training, and dance as a part of broader programs [25, 27].

Summing up, the findings of the present study add further support to the hypothesis of a positive effect of PA on work status in cancer populations. Positive associations between PA and work have been found in non-cancer populations as well, further supporting the hypothesis [67, 68]. Furthermore, by adding a broader concept of PA, i.e., PA_daily,_ the present study contributes with new knowledge. The previous studies within this field, the observational [23, 24] as well as the controlled studies [4, 20–22, 25–28], all examined the effect of PA interventions or PA as sports/exercise. Defining PA as including all daily activity is in accordance with the understanding of PA as *“any bodily movement produced by skeletal muscles”* as defined by the World Health Organization [69]. The numerous health benefits of an active life style have been well known for several years [69, 70]. The present study adds to existing evidence that these benefits may also apply to the work lives of cancer patients. Furthermore, when measuring the effect of PA by means of controlled studies including an intervention program, the specific effects of PA are difficult to conclude upon. The previous controlled studies within the area, in which the positive effect of PA was confirmed, included PA programs consisting of PA sessions supervised by specially trained physical therapists [4, 20–22] and in several cases including individual coaching as well [4, 22]. Hence, in these trials, the observed effects may not be attributed solely to the level of PA but to participating in an intervention, getting personal supervision and coaching as well. The findings of the present study add to existing evidence, that the effect of PA on work status seems independent of participation in a PA program, i.e., the positive effect appears to be related to the PA and not mainly to the effects of being in a PA intervention program. Thus, facilitating an active life style, either by PA intervention programs or by other means, appears to positively affect the working lives of employees undergoing treatment for cancer.

#### The mediating role of RTWSE (objective III)

A Danish validated scale was used to measure RTWSE [59] and the hypothesis regarding the mediating role of RTWSE was based on previous research. Yet, the results failed to support the hypothesis of RTWSE being a mediator of the observed association between PA and work status. However, it should be recognized that the ORs in the mediation models are in the expected direction and well above 1.00, yet not significant. Due to a small sample size in the present study, the risk of type II is present, and the mediating role of RTWSE should not be rejected solely on the present results. Future studies with larger sample sizes examining the mediating role of RTWSE in the association between PA and work status in employees with cancer are thus recommended.

Alternative mediators should be examined, e.g., improved fitness or renewed energy as reported in a qualitative study by Groeneveld et al. [19] and supported in an RCT by Van Waart et al. [20], in which the participants in the control group (i.e., care as usual) reported physical health limitations as the reason for not returning to work. Reduced fatigue is another possible mediator. Fatigue has shown to be reduced by exercise [71] and further to be associated with later RTW and reduced work ability [13, 49, 72]. By reducing fatigue, PA may facilitate RTW and increase work ability in employees with cancer. In future research, it is recommended examining fatigue as a potential mediator.

### Strengths and limitations

A strength of the present study is the use of register-based information to measure the dependent variable, i.e., work status, allowing a 100% follow-up. Several limitations should be noted. First, our analysis of potential selection bias is limited. It was not possible to obtain information regarding the large group of patients who did not return the contact sheet at Aarhus University Hospital (*n* = 416). Hence, comparisons between the responders and the non-responders with regard to significant sociodemographic and illness- and treatment-related variables were impossible but could have informed about selection bias. In general, social inequality is well documented regarding participation in projects [73]. It has been found that the non-responders of cancer studies were less likely to be working and that they tended to have a lower education than participants [20, 24]. Hence, the present study may not be generalizable to the less educated employees with cancer. Secondly, all participants received chemotherapy, hence these results may not be generalizable to cancer patients who do not receive chemotherapy but only other cancer treatments, e.g., immune therapy, radiotherapy, or surgery. Further, due to missing data regarding employment status and language skills of the non-responders, the exact number of eligible participants are unknown and the exact participation rate therefore remains unknown. Similar limitations have been reported in other studies within this area [35].

Furthermore, the small number of participants increased the risk of overfitting the statistical models. However, based on multiple analyses, Vittinghoff et al. [74] suggested that the number of minimum events per explanatory parameter can be reduced to 5–9 events, as in the present study, without increasing the risk of misinterpretation of the results considerably. However, future studies with larger samples are recommended. A larger sample would further have allowed for including more covariates in the statistical models. Several additional illness- and treatment-related variables would have been relevant to include, e.g., diagnosis, number of treatment modalities, cancer stage, and other indicators of disease severity which have proven to be associated with RTW in cancer populations [12, 13].

A final limitation is the measurement of PA by self-report. Using questionnaires to measure PA is a common method, but the participants may have overestimated own level of PA as PA is a socially desirable behavior [75]. Using electronic devices to determine the level of PA, i.e., accelerometers and heart rate monitors, could have been a more objective assessment method. The reported level of current PA may furthermore have been influenced by treatment-related side effects, as the seven-day period in which the participants were told to rate their current level of PA, could be immediately after the date of chemotherapy infusion. This could affect the PA level reported. The reported level would thus not be an estimate of PA in general. We did not explore this and the data was therefore not available to us, which could be a study limitation.

## Conclusion

PA appears positively associated with work status in employees undergoing treatment for cancer in the twelve months period after initiating chemotherapy. The hypothesis of RTWSE being the mediator between PA and work was not confirmed. Future studies with larger sample sizes examining the mediating role of RTWSE as well as the mediating role of other variables, i.e., fatigue, renewed energy, and increased physical function in the observed association between PA and work status in employees with cancer are recommended. Further, future research would benefit from examining the significance of ongoing PA and changes in PA patterns in relation to work status, work ability and RTW in populations of employees with cancer.

## Data Availability

The datasets generated during and/or analyzed during the current study are available from the corresponding author on reasonable request.

## Abbreviations

Current PA_daily_: The current level of daily physical activity
Current PA_leisure_: The current level of leisure time physical activity
DREAM: Danish Register for Evaluation of Marginalization
IQR: Interquartile range
OR: Odds ratio
PA: Physical activity
Pre-illness PA_leisure_: The pre-illness level of leisure time physical activity
RCT: Randomised Controlled Trial
RTW: Return to work
RTWSE: Return to work self-efficacy
SE: Self-efficacy
SD: Standard Deviations
